# From Guidelines to Clinical Practice: Expert Perspectives on Long‐Term Management of Obesity

**DOI:** 10.1002/oby.70263

**Published:** 2026-08-26

**Authors:** Hong Chen, Ling Li, Junqing Zhang, Shaihong Zhu, Marc‐André Cornier, Jonathan Q Purnell

**Affiliations:** ^1^ Zhujiang Hospital of Southern Medical University Guangzhou China; ^2^ Zhongda Hospital, Southeast University Nanjing China; ^3^ Peking University First Hospital Beijing China; ^4^ Division Endocrinology, Diabetes and Metabolic Diseases, Department of Medicine The Third Xiangya Hospital of Central South University Changsha China; ^5^ Division of Endocrinology, Diabetes & Metabolic Diseases, Department of Medicine Medical University of South Carolina Charleston South Carolina USA; ^6^ Department of Medicine Oregon Health & Science University Portland Oregon USA

**Keywords:** GLP‐1 RA, GLP‐1/GIP dual RA, long‐term management, obesity

## Abstract

An international panel of experts from the U.S. and China convened to discuss the *2026* *TOS–OMA–OAC Expert Guidance Statement on the Pharmacological Management of Obesity,* focusing on emerging evidence, regional considerations, and challenges in translating recommendations into long‐term care. Panelists reached a consensus that obesity should be recognized as a chronic and progressive disease, for which pharmacotherapy is lifelong once initiated, as discontinuation consistently leads to weight regain and loss of metabolic benefit. The discussion highlighted a shift toward treating obesity itself as an upstream intervention capable of modifying multiple obesity‐related complications, with greater weight loss generally conferring greater clinical benefit. Visceral adipose tissue emerged as a particularly relevant therapeutic target, especially in Asian populations, where substantial metabolic risk may be present at lower BMI levels. Panelists noted heterogeneity in treatment response and that eventual weight‐loss plateaus should be anticipated as evidence of maximal effectiveness rather than medication failure. The panel endorsed an individualized, stepwise approach integrating pharmacotherapy and metabolic surgery as complementary strategies. Finally, the panel emphasized that translating therapeutic advances into real‐world benefit will require addressing practical barriers related to policy, access, affordability, and stigma to ensure effective care reaches those who need it most.

## 1 Introduction

Obesity is a major global health challenge affecting over 890 million adults worldwide [1]. According to an analysis from the Global Burden of Disease Study, without urgent action, over half the world's adult population is forecast to be living with overweight or obesity by 2050 [2]. Obesity is causative of, or a major risk factor for, many noncommunicable diseases such as cardiovascular diseases, type 2 diabetes (T2D), cancer, obstructive sleep apnea (OSA), metabolic dysfunction–associated steatotic liver disease/metabolic dysfunction–associated steatohepatitis (MASLD/MASH), polycystic ovary syndrome, osteoarthritis (OA), and chronic kidney disease. The burden of disease attributable to obesity has risen sharply over the past three decades, with both mortality and disability‐adjusted life years increasing substantially since 1990 and projected to continue rising [3]. In 2021 alone, obesity is estimated to have caused 3.7 million deaths from noncommunicable diseases [1]. Despite the World Health Organization (WHO) recognizing obesity as a chronic disease requiring long‐term care, current clinical practice often falls short of this standard. Obesity is still frequently approached as a result of individual lifestyle choice or a modifiable risk factor rather than a disease in its own right, leading to stigma, delayed diagnosis, inadequate treatment, and related comorbidities [4].

Until recently, pharmacological options for obesity management were limited by modest efficacy and poor tolerability, along with a lack of robust evidence supporting long‐term efficacy and benefits beyond weight loss [5]. This paradigm has shifted with the emergence of highly effective therapies, particularly glucagon‐like peptide‐1 (GLP‐1) receptor agonists and GLP‐1/glucose‐dependent insulinotropic polypeptide (GIP) dual receptor agonists [6]. Long‐term studies have provided evidence of sustained weight loss, favorable safety profiles, and metabolic and cardiovascular benefits, supporting the use of these newer pharmacotherapies for chronic obesity management [7, 8, 9, 10, 11]. In this context, updated clinical guidance is urgently needed to support evidence‐based implementation of these therapies. In 2025, the European Association for the Study of Obesity published a comprehensive obesity management framework emphasizing a multidisciplinary, patient‐centered approach [12]. In addition, WHO recently released global guidelines on the use of GLP‐1 receptor agonist medicines for obesity treatment, providing evidence‐informed recommendations on GLP‐1 receptor agonists and GLP‐1/GIP dual agonists as part of chronic care for adults living with obesity and obesity‐related complications [1]. Yet the most recent comprehensive obesity management guidelines in the U.S. were published in 2015 [13]. These guidelines recommended a stepwise approach beginning with lifestyle interventions [14, 15], followed by pharmacotherapy for individuals with a body mass index (BMI) ≥ 30 or ≥ 27 kg/m^2^ with weight‐related comorbidities, and framed obesity management largely around optimizing care for obesity‐related complications. However, the rigid tiered structure of these stepwise pathways requires patients to exhaust behavioral therapy before accessing pharmacotherapy, potentially delaying or preventing access to effective treatment and allowing the progression and worsening of obesity‐related comorbidities [16]. The profound transformation in the obesity pharmacotherapy landscape since 2015 underscores the need to revisit existing U.S. obesity management recommendations.

In response to this pressing need, three leading U.S. obesity‐focused organizations, The Obesity Society (TOS), the Obesity Medicine Association (OMA), and the Obesity Action Coalition (OAC), jointly developed the *2026 TOS–OMA–OAC Expert Guidance Statement on the Pharmacological Management of Obesity* [17]. Each organization brings a distinct perspective: TOS focuses on translating scientific evidence into comprehensive obesity care, OMA emphasizes the delivery of obesity care by healthcare providers, and OAC represents the voices of patients with lived experience with obesity and the provider community, ensuring that patient perspectives directly inform guideline development. As a result, patient quality of life was elevated as a priority outcome. At the Best of ObesityWeek 2025 in China, Professor Jonathan Q Purnell, MD, from Oregon Health & Science University, USA, presented key highlights from this forthcoming statement.

## 2 Talk Summary: U.S. Guideline Highlights and the Challenges in Translating Them Into Practice

The *2026 TOS–OMA–OAC Expert Guidance Statement* [17] was developed using a rigorous, evidence‐based framework to systematically evaluate the available data and to support the implementation of recommendations aimed at optimizing patient health and well‐being. A key additional objective was to address an important practice gap in the U.S.: several obesity medications recently approved by the U.S. Food and Drug Administration (FDA) had not previously been subjected to formal, guideline‐level evaluation. Accordingly, the guidance development process focused on assessing their clinical efficacy, safety, and appropriate role in care while promoting shared decision‐making between healthcare providers and patients.

The recommendations focus on adults and address six FDA‐approved obesity medications, as well as obesity‐related complications where evidence supports improvement with obesity treatment, including T2D, OSA, MASLD/MASH, OA, atherosclerotic cardiovascular disease, and heart failure with preserved ejection fraction (HFpEF). Notably, this guidance is the first to elevate quality of life as a critical outcome measure and to address weight maintenance as a distinct and essential phase of obesity care. Fifteen key clinical questions related to obesity pharmacotherapy were identified and evaluated using the Grading of Recommendations Assessment, Development and Evaluation framework, with explicit consideration of equity, access, and policy implications (Table 1).

**TABLE 1:** Recommendations on pharmacotherapy for the management of obesity in adults 18 years of age or older with either obesity or overweight plus at least one obesity‐related complication.

Recommendation	Strength of recommendation	Quality of evidence
Orlistat should be used compared to placebo.	Conditional	Low
Bupropion‐naltrexone should be used compared to placebo.	Strong	Moderate
Phentermine should be used compared to placebo.	Conditional	Low
Phentermine‐topiramate should be used compared to placebo.	Conditional	Low
Liraglutide should be used compared to placebo.	Conditional	Low
Semaglutide should be used compared to placebo.	Strong	Moderate
Tirzepatide should be used compared to placebo.	Strong	Moderate
Setmelanotide should be used compared to usual care for approved monogenic obesity syndromes (e.g., risk alleles for LEPR, POMC, PCSK1, and BBS).	Strong	Moderate
For those who are engaged in medical obesity treatment (obesity medication) and are in the weight maintenance phase, obesity medication interventions should be used compared to no obesity medication intervention.	Strong	Moderate
For those with obstructive sleep apnea, GLP‐1/GIP receptor agonists should be used compared to placebo.	Conditional	Low
For those with heart failure with preserved ejection fraction, GLP‐1 (+) agonists should be used compared to placebo.	Conditional	Low
For those with metabolic dysfunction–associated steatotic liver disease or metabolic dysfunction–associated steatohepatitis, GLP‐1 (+) agonists should be used compared to placebo.	Conditional	Low
For those with osteoarthritis, GLP‐1 (+) agonists should be used compared to placebo.	Conditional	Low
For those with a history of myocardial infarction, stroke, or symptomatic peripheral vascular disease, semaglutide is better than placebo in reducing major adverse cardiovascular events (myocardial infarction, stroke, and death from myocardial infarction).	Conditional	Low
For those with type 2 diabetes, FDA‐approved obesity medications should be used compared to placebo.	Conditional	Low

*Note:* Strong certainty: We are very confident that the true effect lies close to that of the estimate of the effect; Moderate certainty: We are moderately confident in the effect estimate: the true effect is likely to be close to the estimate of the effect, but there is a possibility that it is substantially different; Low certainty: Our confidence in the effect estimate is limited: the true effect may be substantially different from the estimate of the effect.

Abbreviations: BBS, Bardet–Biedl syndrome; FDA, U.S. Food and Drug Administration; GIP, glucose‐dependent insulinotropic polypeptide; GLP‐1, glucagon‐like peptide‐1; LEPR, leptin receptor; PCSK1, proprotein convertase subtilisin/kexin type 1; POMC, proopiomelanocortin.

### 2.1 Summary of Recommendations

A fundamental shift in treatment philosophy distinguishes the *2026 TOS–OMA–OAC Expert Guidance Statement* [17] from the 2015 guidelines. The 2015 guidelines framed obesity management primarily around optimizing care for obesity‐related comorbidities, with attention to the weight effects and safety of therapeutic interventions. For example, in patients with obesity and T2D requiring insulin, the 2015 guidelines recommended adjunctive metformin, pramlintide, or GLP‐1 receptor agonists to mitigate insulin‐associated weight gain, reflecting a comorbidity‐centered approach in which weight management was secondary [13]. The *2026 TOS–OMA–OAC Expert Guidance Statement*, by contrast, reframes obesity itself as the primary therapeutic target, emphasizing that treating obesity is central to improving downstream complications. Accordingly, the guidance statement issues strong recommendations for obesity medication use while providing conditional recommendations for managing obesity‐related complications, including T2D. This represents a conceptual transition from treating complications with weight awareness to treating obesity as the disease driving those complications using rigorous rating criteria that are becoming widely adopted [1].

Consistent with this reframed treatment paradigm, the *2026 TOS–OMA–OAC Expert Guidance Statement* issues strong recommendations for setmelanotide, bupropion‐naltrexone, semaglutide, and tirzepatide for obesity management. For patients receiving obesity medications who have reached the weight maintenance phase following initial weight loss, continued obesity medication is strongly recommended. The Guidance Statement issues conditional recommendations for phentermine‐topiramate, orlistat, liraglutide, and phentermine. In contrast, conditional recommendations are issued for the treatment of obesity‐related complications, including OSA, HFpEF, MASLD/MASH, OA, major adverse cardiovascular events, and T2D. The Guidance Statement does not provide formal recommendations regarding co‐management with intensive behavioral therapy or structured physical activity interventions or the need for supplemental protein due to insufficient evidence (Table 1).

### 2.2 Translating Guidelines Into Clinical Practice: Principles Over Algorithms

Obesity guidelines from different regions reflect distinct narrative perspectives shaped by healthcare policies, population characteristics, and clinical practice. For example, the 2024 Chinese National Health Commission guidelines for the clinical management of obesity adopt a comprehensive chronic disease management framework tailored to the Chinese population and healthcare context, integrating lifestyle intervention, pharmacotherapy, surgical management, and traditional Chinese medicine across the full spectrum of care [18]. By comparison, Obesity Canada [19] and the European Association for the Study of Obesity [20] provide step‐by‐step decision tools and treatment algorithms for the pharmacological management of obesity, reflecting a more algorithm‐driven approach to clinical decision‐making. A detailed side‐by‐side comparison of the Chinese and European guidelines has been published previously [21]. In this context, the *2026 TOS–OMA–OAC Expert Guidance Statement* [17] adopts a distinct approach to clinical implementation by prioritizing treatment principles over prescriptive algorithms. This decision reflects the recognition that many medication‐selection decisions rely on observational data and expert consensus rather than randomized controlled trials, rendering rigid algorithms potentially limiting across diverse clinical contexts. Instead, Professor Purnell highlighted the basic and advanced principles of patient management that guide clinical practice.

First and foremost, obesity should be recognized and managed as a chronic disease. This framing has important clinical implications, particularly for expectations around long‐term management. Lifestyle interventions, including healthy eating and regular physical activity, remain foundational but are frequently insufficient on their own to achieve meaningful and sustained weight loss. Long‐term studies conducted under optimal conditions show an average of 2%–4% total weight loss with lifestyle interventions alone [14, 15], highlighting that weight regain is largely driven by biological mechanisms rather than lack of adherence or willpower. In particular, weight loss triggers coordinated adaptations in appetite regulation and energy expenditure that promote weight regain. Appetite is regulated by multiple circulating hormones. One, ghrelin, stimulates hunger, whereas several others are anorexigenic, including leptin, peptide YY, cholecystokinin, insulin, amylin, pancreatic polypeptide, and GLP‐1. Following diet‐induced weight loss, ghrelin levels increase whereas anorexigenic hormone levels decrease, and these changes persist for at least 1 year, producing a sustained biological drive to regain weight [22]. In parallel, resting energy expenditure decreases by approximately 500 kcal/day beyond what would be predicted based on body mass loss, an adaptation that can persist for up to 6 years after initial weight loss [23]. Pharmacotherapy therefore plays a critical role by targeting key central nervous system pathways regulating counter‐adaptive responses that normally would enhance appetitive behaviors (“food noise”) and lead to positive energy balance [24]. Accordingly, obesity pharmacotherapy should be intended for long‐term use once initiated, except in specific circumstances such as intolerable adverse effects, pregnancy, or the availability of more effective therapies.

Building on these core principles, Professor Purnell highlighted several advanced considerations for guiding clinical practice. First, patient counseling should emphasize that average group outcomes from published studies do not predict individual response. As in the treatment of other chronic diseases such as hypertension or T2D, weight‐loss response is heterogeneous, ranging from non‐response up to 50% (or more) total weight loss with a given therapy. Therefore, when patients fail to lose weight on any specific obesity medication, this is not a reflection of treatment failure but rather a lack of alignment of that drug's mechanism of action for that individual's biology. As in the management of other chronic diseases, this variability supports the growing role of combination therapy. Second, treatment goals should extend beyond weight loss alone to include broader health outcomes, including quality of life and functional improvement. Another advanced concept discussed was the strategy of treating obesity itself to address obesity‐related complications. Professor Purnell noted that in current clinical practice, when lifestyle modification is insufficient, clinicians often proceed directly to disease‐specific pharmacotherapy for conditions such as diabetes, dyslipidemia, or hypertension. However, an alternative approach is to prioritize obesity treatment, either before or alongside disease‐specific therapies, with the aim of addressing the upstream driver of multiple cardiometabolic conditions. Clinical examples illustrated that even modest‐to‐moderate weight loss achieved through obesity pharmacotherapy can improve glycemic control, lipid profiles, and blood pressure, potentially reducing the need for additional medications. These benefits are often mechanistically linked to reductions in visceral and ectopic fat [25], as excess visceral fat and hepatic lipid accumulation drive insulin resistance, dysglycemia, and mixed hyperlipidemia [26]. By reducing visceral and ectopic fat, obesity treatment can improve insulin sensitivity, lower triglyceride levels, and ameliorate glucose dysregulation, even in individuals who do not have excess adiposity to meet conventional criteria for obesity, which is especially relevant to patients of Asian ancestry (see Section 5) [27]. With nearly 200 recognized obesity‐related complications spanning metabolic, mechanical, and mental health domains [28], effective obesity treatment can generate a broad systemic benefit for overall health.

## 3 Panel Discussion

The panel discussion session was chaired by Professor Marc‐André Cornier, MD, from the Medical University of South Carolina, USA, who also served as President of TOS. Panelists included Professor Hong Chen, MD, PhD, from Zhujiang Hospital of Southern Medical University, China; Professor Jonathan Q Purnell, MD, from Oregon Health & Science University, USA, President‐Elect of TOS; Professor Junqing Zhang, MD, PhD, from Peking University First Hospital, China; Professor Ling Li, MD, PhD, from Zhongda Hospital, Southeast University, China; and Professor Shaihong Zhu, MD, PhD, from the Third Xiangya Hospital of Central South University, China.

### 3.1 Topic 1: Obesity as a Chronic Disease and the Need for Long‐Term Therapy

The panel reached a strong consensus that obesity should be recognized and treated as a chronic disease driven by excess and abnormal adiposity rather than body weight alone and tightly linked to metabolic dysregulation and increased mortality. Lifestyle modifications, while foundational, are difficult to sustain and often inadequate for achieving and maintaining clinically meaningful weight loss on their own. Panelists stressed that obesity pharmacotherapy should be approached similarly to other chronic conditions such as hypertension or diabetes, where treatment is not discontinued once an initial response is achieved. Sustained therapy is essential for maintaining clinical benefits, as treatment discontinuation is consistently associated with weight regain and reversal of cardiometabolic improvements. A post hoc analysis of the SURMOUNT‐4 trial provided compelling evidence: in adults with obesity who achieved ≥ 10% weight loss by week 36 on tirzepatide and were then switched to placebo (*n* = 308), greater weight regain over weeks 36–88 was associated with a dose–response reversal of cardiometabolic improvements: systolic blood pressure increased by 6.8–10.4 mmHg, non‐high‐density lipoprotein cholesterol increased by up to 10.8%, and HbA1c increased by 0.14%–0.35%. In addition, compared with a waist circumference increase of 14.7 cm in participants with ≥ 75% weight regain, those with < 25% weight regain had an increase of only 0.8 cm, which is unlikely to meaningfully affect risk factor recurrence [29]. These findings demonstrate that maintenance therapy is critical to preserving weight loss and sustained metabolic benefits in obesity pharmacotherapy. In this context, oral GLP‐1 receptor agonists represent an emerging option for sustained weight maintenance, as they require no injection devices or cold chain, are simpler and less costly to manufacture, and are more readily deployed in primary care. The first oral GLP‐1 receptor agonist approved for chronic weight management is oral semaglutide. The approval was supported by phase 3 OASIS 4 data demonstrating an estimated 16.6% total weight loss, a favorable safety profile, and improvements in cardiometabolic risk factors [30]. Additionally, a recently FDA‐approved oral non‐peptide GLP‐1 receptor agonist [31] demonstrated adequate weight‐loss maintenance (< 20% regain) at 52 weeks in the phase 3 ATTAIN‐MAINTAIN study among patients transitioned from injectable GLP‐1 receptor agonists (tirzepatide or semaglutide) [32]. Oral formulations may offer adherence and affordability advantages over injectables in long‐term maintenance settings, supporting a potential treatment strategy in which oral agents facilitate sustained weight maintenance following injectable GLP‐1–induced weight loss [33].

Chinese experts advocated for a personalized, long‐term treatment approach in which pharmacotherapy and surgery are positioned as complementary rather than as competing strategies, with treatment decisions guided by ongoing patient–clinician dialogue. For example, for patients reluctant to undergo bariatric surgery, a 2– to 3‐month trial of GLP‐1‐based pharmacotherapy can identify early responders who may achieve meaningful weight loss without surgery. Conversely, those with very high BMI and inadequate pharmacotherapy response may require surgical intervention. In severe obesity, pharmacotherapy can reduce preoperative surgical and anesthesia risk. Postoperatively, GLP‐1 therapy may serve as salvage treatment for weight regain when repeat surgery is not desired. The panelists viewed the safety profile of current obesity pharmacotherapies favorably during long‐term treatment.

### 3.2 Topic 2: Regional Differences and the Asian Phenotype of Visceral Adiposity

In the panel discussion, experts emphasized that Asian populations have distinct adiposity characteristics: higher body fat percentage and greater visceral adipose tissue (VAT) at lower BMI levels, leading to increased metabolic risk even at “normal” BMI. This is reflected in the lower BMI cutoffs adopted by WHO for defining overweight and obesity in Asian populations [34]. Chinese experts highlighted that VAT is more clinically relevant than subcutaneous fat in Asian populations, and early detection of visceral adiposity was considered essential for timely intervention and prevention of downstream metabolic and cardiovascular complications. Data from the SELECT trial suggest Asian populations may derive greater cardiovascular benefit despite lower baseline BMI, reinforcing visceral adiposity as a key risk stratification marker where BMI alone is insufficient [35]. Since BMI alone may underestimate cardiometabolic risk, panelists recommended a multidimensional assessment strategy including waist circumference, waist to hip or waist to height ratios, and imaging‐based assessment of fat distribution, as well as laboratory markers for liver fibrosis, insulin resistance, mixed hyperlipidemia, uric acid, and high‐sensitivity C‐reactive protein [36]. Although the 2024 Chinese National Health Commission guidelines specify BMI‐based thresholds for initiating obesity pharmacotherapy, accumulating evidence that metabolic complications can arise at lower BMI in Asian populations supports ongoing refinement of these criteria to incorporate measures of visceral adiposity and overall cardiometabolic risk, as endorsed by both Chinese [18] and international guidance.

From a therapeutic perspective, Chinese experts noted that medication selection should account for overall efficacy, differential effects on lean versus fat mass, and specific impacts on VAT and central obesity. When greater fat reduction is required, tirzepatide was cited as a preferred option based on its superior effects on body weight and fat loss. The SURPASS‐3 MRI substudy demonstrated that tirzepatide significantly reduced liver fat content and visceral and abdominal subcutaneous adipose tissue volumes compared with insulin degludec in patients with T2D [37]. Tirzepatide also showed potentially favorable changes in muscle fat infiltration, with muscle volume reductions proportional to overall weight loss, supporting its use in achieving substantial fat loss while preserving lean mass [38].

Although growing evidence supports ethnic differences in adiposity distribution and metabolic risk, the panel highlighted that Asian participants have historically been underrepresented in large multinational studies, potentially limiting the generalizability of treatment effects across populations. Recent clinical trials have begun to address this gap through region‐specific studies. For example, SURMOUNT‐CN and SURMOUNT‐J are designed to generate country‐specific evidence for the efficacy and safety of tirzepatide in China and Japan [39, 40]. In addition, the STEP program was expanded to include regional and country‐specific studies evaluating semaglutide in Asian populations, including trials conducted across East Asia (STEP 6 and STEP 7) and in South Korea and Thailand [41, 42, 43]. Mazdutide, a dual glucagon/GLP‐1 receptor agonist, has also been evaluated in Chinese adults with overweight or obesity [44]. Data from these trials are essential for tailoring treatment strategies to the unique metabolic characteristics of Asian individuals and for ensuring that guideline recommendations are applicable across diverse populations.

### 3.3 Topic 3: Heterogeneity of Treatment Response

Panelists shared their clinical experience and agreed that heterogeneity in response to obesity treatment is common and reflects variation in disease severity, underlying biology, drug effectiveness, number and severity of comorbidities, and patient preferences. With newer obesity medications, patients achieve an initial response within the first 3 to 6 months of treatment, with weight loss often reaching a plateau between 6 and 12 months. This trajectory raises a common clinical dilemma regarding whether therapy should be continued or discontinued once weight loss stabilizes. The discussion highlighted concerns that early discontinuation, even after an apparently strong initial response, may lead to significant weight regain, in some cases exceeding baseline body weight. Therefore, the panel supported continued treatment beyond the plateau phase, emphasizing that ongoing therapy can help sustain broader metabolic benefits, including improvements in dyslipidemia, fatty liver disease, and cardiovascular risk factors.

The panel acknowledged that important evidence gaps remain. In particular, the optimal maintenance dose and duration of pharmacotherapy required to preserve long‐term treatment response have yet to be clearly defined and represent key priorities for future research. Beyond these evidence gaps, substantial real‐world implementation challenges persist. Despite the clear rationale for long‐term therapy, significant practical barriers limit access, including high costs and restrictive insurance coverage. In some settings, including China, limited drug availability adds to these constraints, as several newer agents have not yet entered the domestic market or been included in national reimbursement schemes. These practical barriers are further compounded by sociocultural factors. Growing evidence indicates that societal perceptions of body size, stigma surrounding obesity medications, and insufficient recognition of obesity as a chronic disease can reduce patients' willingness to seek care and undermine long‐term treatment adherence. Addressing these sociocultural barriers has become a policy priority. For instance, the 2024 Japanese obesity management guidelines explicitly emphasize that eliminating obesity‐related stigma is essential for improving patient care and quality of life [45]. Similarly, China launched a 3‐year weight management initiative in 2024, focusing on widespread adoption of healthy lifestyle behaviors and sustained weight management at the population level [46].

### 3.4 Topic 4: Obesity‐Related Complications and the Need for Timely Intervention

Panelists identified a critical barrier: the need for a fundamental shift in clinical mind‐set by many, if not most, practitioners. Obesity should be managed like other chronic diseases, with treatment goals focused on long‐term risk reduction rather than short‐term weight loss. Current clinical practice remains largely “function/parameter‐driven,” focusing primarily on isolated metrics such as weight or BMI, which fail to capture the full spectrum of obesity‐related health impacts. Importantly, many obesity‐related complications evolve over time and may become only partially reversible once established, meaning that delaying intervention can limit the opportunity to modify long‐term metabolic and cardiovascular risk. As seen in other chronic conditions such as T2D, timely and sustained control of obesity may confer durable protection against long‐term complications [47]. However, due to healthcare resource limitations and patient expectations, current practice predominantly emphasizes short‐term weight reduction over long‐term management, with many patients using weight‐loss medications solely for weight reduction rather than sustained health benefits. The panel emphasized that obesity care needs to evolve toward holistic management that addresses the underlying disease and its multiple manifestations.

From both U.S. and Chinese perspectives, the panel therefore framed obesity treatment as an upstream intervention capable of mitigating the development and progression of multiple obesity‐related complications, rather than merely a strategy for weight reduction (Table 2). For example, in adults with prediabetes and overweight or obesity, the 3‐year SURMOUNT‐1 study showed that once‐weekly tirzepatide reduced the risk of progression to T2D by 94% compared with placebo [51], demonstrating that obesity treatment can modify long‐term metabolic risk beyond weight reduction alone. The panel further discussed the relationship between the magnitude of weight loss and graded clinical benefits (Table 3). Weight loss exceeding 5% was associated with improvements in multiple metabolic and some nonmetabolic outcomes, while reductions greater than 10% conferred additional benefits in glycemic control, blood pressure, and lipid profiles. Weight loss exceeding 15% was associated with more pronounced cardiovascular benefit, underscoring the relevance of achieving and sustaining larger degrees of weight reduction in selected patients. While these data highlight the value of substantive weight reduction, the panel cautioned that prioritizing obesity as an upstream therapeutic target does not imply deferring guideline‐directed medical therapy for established comorbidities. In patients with obesity and poorly controlled hypertension, dyslipidemia, or hyperglycemia, obesity pharmacotherapy and disease‐specific therapies should be initiated concurrently, as adequate blood pressure, lipid, or glycemic control may not be fully achievable through obesity treatment alone or may require a prolonged treatment course. Consistent with this principle, the 2024 Chinese National Health Commission guidelines recommend that in patients with obesity‐related hypertension, pharmacological management should simultaneously address weight control and associated metabolic dysregulation alongside blood pressure reduction, supporting an integrated rather than sequential treatment approach [18].

**TABLE 2:** Incretin‐based pharmacotherapy trials with obesity‐related complications as primary endpoints.

Study	Study design	Study population	Primary endpoints	Key findings
SELECT trial (semaglutide 2.4 mg) [9]	Randomized, double‐blind, placebo‐controlled phase 3 trial	17,604 adults with overweight or obesity and established CVD, without diabetes	MACE: cardiovascular death, nonfatal myocardial infarction, nonfatal stroke	20% reduction in MACE risk (HR 0.80, 95% CI 0.72–0.90, *p* < 0.001)
ESSENCE trial (semaglutide 2.4 mg) [10]	Randomized, double‐blind, placebo‐controlled phase 3 trial; total duration 240 weeks; prespecified interim analysis at week 72 (first 800 participants)	1197 patients with biopsy‐confirmed MASH and stage F2–F3 liver fibrosis	Resolution of steatohepatitis without worsening of fibrosis; improvement in fibrosis without worsening of steatohepatitis	Steatohepatitis resolution without fibrosis worsening: 62.9% vs. 34.3% (*p* < 0.001) Fibrosis improvement without steatohepatitis worsening: 36.8% vs. 22.4% (*p* < 0.001) Both endpoints achieved: 32.7% vs. 16.1% (*p* < 0.001) Body weight change: −10.5% vs. −2.0% (*p* < 0.001)
SYNERGY‐NASH trial (tirzepatide 15 mg) [11]	Randomized, double‐blind, placebo‐controlled phase 2 trial (52 weeks)	Patients with MASH and moderate or severe liver fibrosis	Resolution of MASH without worsening of fibrosis	MASH resolution: 62% vs. 10% (*p* < 0.001) ≥ 1‐stage fibrosis improvement without worsening: 51%
SURMOUNT‐OSA trial (tirzepatide 10/15 mg) [48]	Randomized, double‐blind, placebo‐controlled phase 3 trial (52 weeks)	Adults with moderate‐to‐severe OSA and obesity; baseline mean AHI 50 events/h; mean BMI = 39 kg/m^2^	Change in AHI at week 52	No PAP: AHI change −25.3 vs. −5.3 events/h (*p* < 0.001) Receiving PAP: AHI change −29.3 vs. −5.5 events/h (*p* < 0.001) Significant reductions in body weight, hypoxic burden, hsCRP, and systolic blood pressure, with improvements in sleep‐related patient‐reported outcomes
SURPASS‐CVOT trial (tirzepatide up to 15 mg) [49]	Randomized, double‐blind, active‐comparator–controlled, noninferiority trial (median follow‐up 4.0 years)	13,299 patients with type 2 diabetes and atherosclerotic CVD	A composite of death from cardiovascular causes, myocardial infarction, or stroke (tested for noninferiority vs. dulaglutide 1.5 mg)	Tirzepatide was noninferior to dulaglutide: primary endpoint occurred in 12.2% vs. 13.1% (tirzepatide vs. dulaglutide); HR 0.92 (95.3% CI 0.83–1.01); *p* = 0.003 for noninferiority; *p* = 0.09 for superiority
TRIUMPH‐4 trial (retatrutide 12 mg) [50]	Randomized, double‐blind, placebo‐controlled phase 3 trial (68 weeks)	Adults with overweight or obesity and knee osteoarthritis	Percent change in body weight at week 68; change in WOMAC pain score	Percentage change in body weight: −26.4% (−29.1 kg; 9 mg), −28.7% (−32.3 kg; 12 mg), and −2.1% (−2.1 kg; placebo) Change in WOMAC pain subscale score: −4.5 points (−75.8%; 9 mg), −4.4 points (−74.3%; 12 mg), and −2.4 points (−40.3%; placebo)

Abbreviations: AHI, apnea–hypopnea index; CVD, cardiovascular disease; HR, hazard ratio; hsCRP, high‐sensitivity C‐reactive protein; MACE, major adverse cardiovascular events; MASH, metabolic dysfunction–associated steatohepatitis; OSA, obstructive sleep apnea; PAP, positive airway pressure; WOMAC, Western Ontario and McMaster Universities Osteoarthritis Index.

**TABLE 3:** Weight‐loss responses with tirzepatide, semaglutide, and retatrutide across phase 3 trials.

	Retatrutide (GIP/GLP‐1/glucagon)		Tirzepatide (GIP/GLP‐1)	Semaglutide (GLP‐1)
Clinical trial	TRIUMPH‐4 (68 weeks)^a^		SURMOUNT‐5 (72 weeks)^b^	
	9 mg	12 mg		
Mean weight loss	26.4%	28.7%	20.2%	13.7%
≥ 10% weight loss	/	/	81.6%	60.5%
≥ 15% weight loss	/	/	64.6%	40.1%
≥ 20% weight loss	/	/	48.4%	27.3%
≥ 25% weight loss	47.7%	58.6%	31.6%	16.1%
≥ 30% weight loss	30.5%	39.4%	19.7%	6.9%
≥ 35% weight loss	18.2%	23.7%	/	/

Abbreviations: GIP, glucose‐dependent insulinotropic polypeptide; GLP‐1, glucagon‐like peptide‐1.

^a^ TRIUMPH‐4: adults with overweight or obesity and knee osteoarthritis, retatrutide (9 or 12 mg) once weekly.

^b^ SURMOUNT‐5: adults with obesity but without type 2 diabetes, tirzepatide (10 or 15 mg) or semaglutide (1.7 or 2.4 mg) once weekly.

Severe obesity, particularly at BMI levels exceeding 50 kg/m^2^, was recognized as a distinct clinical context. Panelists noted that such patients often progress from metabolically healthier states to metabolically unhealthy obesity, frequently accompanied by MASLD/MASH, insulin resistance, and vascular disease [7, 8]. In this population, lifestyle intervention alone was considered insufficient, and combined strategies incorporating surgery, pharmacotherapy, and behavioral or psychological support were viewed as necessary to achieve meaningful benefits. Finally, the panel emphasized that the interaction and connectivity between the gut and brain play a central role in energy balance, appetite regulation, and obesity‐related behaviors. Understanding and targeting this bidirectional axis was considered fundamental to the development of more effective, mechanism‐based obesity treatments.

## 4 Overall Panel Consensus

The *2026 TOS–OMA–OAC Expert Guidance Statement* [17] represents a paradigm shift in obesity management, reframing obesity as the primary therapeutic target rather than a secondary consideration in comorbidity management. By issuing strong recommendations for obesity medications while providing conditional recommendations for obesity‐related complications, the Guidance Statement positions obesity treatment as an upstream intervention capable of modifying multiple obesity‐related complications.

Through the integration of real‐world clinical experience from both Asian and Western experts, the panel discussion underscores practical considerations that are central to translating guideline recommendations into routine care, with the aim of supporting more individualized obesity management across heterogeneous healthcare settings. These considerations included decision‐making around long‐term pharmacotherapy, treatment discontinuation, and transitions across therapeutic strategies, and they informed the panel's consensus on several key principles. First, obesity must be recognized and treated as a chronic disease, with treatment goals extending beyond weight loss to include broader cardiometabolic benefits and quality of life improvements. Obesity pharmacotherapy, once initiated, is intended for long‐term or lifelong use. Second, population‐specific considerations, particularly the Asian phenotype of visceral adiposity at lower BMI levels, necessitate comprehensive assessment beyond BMI alone and support earlier intervention in at‐risk populations. Third, the heterogeneity of treatment response and common occurrence of weight‐loss plateaus underscore the need for individualized, stepwise care integrating both pharmacotherapy and metabolic surgery as complementary rather than competing options.

While the evidence base and therapeutic options for obesity have advanced substantially, the panel emphasized that clinical impact will ultimately depend on effective implementation within healthcare systems. Persistent barriers, including limited access, affordability, stigma, and gaps in clinician and patient education, must be addressed. Overcoming these challenges will require coordinated action among healthcare providers, payers, policy makers, and patient advocacy groups to ensure that advances in obesity pharmacotherapy translate into meaningful and equitable improvements in care for those who need it most.

## Funding

This work is derived from a panel discussion within the Best of ObesityWeek 2025 in China program. The program receives an independent medical education grant from Eli Lilly China. The funder has had no influence on the opinions or views expressed herein, and the content remains independent of any commercial interests.

## Conflicts of Interest

Jonathan Q Purnell reported grants from NIH; consulting fees from Novo Nordisk, Regeneron, Boehringer Ingelheim, and Zealand Pharmaceuticals; payment or honoraria from The Obesity Society (TOS); meetings/travel support from TOS; participation on data safety and monitoring board for NIH Metformin in Alzheimer Dementia Prevention; and serving as a TOS Executive Committee member. Marc‐André Cornier reported consulting fees from AstraZeneca, Biophytis, Bonus Health, Enveda, Keros, Eli Lilly, Novo Nordisk, Rivus, Wave, and Zyversa; participation on a data safety and monitoring board or advisory board for Advarra; and serving as President and Past‐President of TOS. Hong Chen, Ling Li, Junqing Zhang, and Shaihong Zhu declare no conflicts of interest.

## Data Availability

Data sharing is not applicable to this article as no datasets were generated or analyzed during the current study.
